# Treatment of a patient with shock complicating severe falciparum malaria: a case report

**DOI:** 10.1186/1757-1626-2-6644

**Published:** 2009-04-20

**Authors:** Friedhelm Kuethe, Ruediger Pfeifer, Silke Rummler, Katharina Bauer, Virginia Kamvissi, Wolfgang Pfister

**Affiliations:** 1 Klinik für Innere Medizin I, Universitätsklinikum der Friedrich-Schiller-Universität, Erlanger Allee 101, 07747 Jena, Germany; 2 Klinik für Innere Medizin I, Universitätsklinikum der Friedrich-Schiller-Universität, Erlanger Allee 101, 07747 Jena, Germany; 3 Institut für Transfusionsmedizin, Universitätsklinikum der Friedrich-Schiller-Universität, Erlanger Allee 101, 07747 Jena, Germany; 4 Institut für Medizinische Mikrobiologie, Universitätsklinikum der Friedrich-Schiller-Universität, Semmelweis-Str. 4, 07743 Jena, Germany; 5 Medizinische Klinik III, Universitätsklinikum der Carl-Gustav-Carus Universität, Fetscherstr. 74, 01307 Dresden, Germany; 6 Institut für Medizinische Mikrobiologie, Universitätsklinikum der Friedrich-Schiller-Universität, Semmelweis-Str. 4, 07743 Jena, Germany

## Abstract

**Introduction:**

Malaria is a potentially life-threatening disease, especially when complicated by a septic shock. When patients present in such a critical condition, the currently available literature allows a dilemma to develop as to which the correct treatment strategy is concerning fluid resuscitation.

**Case presentation:**

A 55-year-old Caucasian man was admitted to the intensive care unit with the clinical picture of severe malaria, brought by a *Plasmodium falciparum* infection. On admission, the patient was confused, had high fever up to 40°C, and his blood analysis revealed a severe thrombocytopenia, a parasitemia of 25.5%, and biochemical features indicative of severe malaria. The patient received quinine and underwent two automated red cell exchanges by use of a centrifuge-driven cell separator. Two days after admission, the patient developed a septic shock. He received an "early-goal" treatment, according to the surviving sepsis campaign guidelines, which propose fluid resuscitation. The existing recommendations concerning the treatment of severe malaria that favour a restrictive fluid administration were disregarded. Fluid therapy was guided by regular measurements of the central venous pressure, blood pressure and monitoring of the hemodynamic status. The patient survived the shock and the subsequent multiorgan failure, which required mechanical ventilation and dialysis. After 12 days in the intensive care unit and an additional three weeks of hospitalization, the patient was discharged to rehabilitation.

**Conclusion:**

The authors believe that in patients with severe malaria complicated by septic shock, the treatment of sepsis and septic shock should be the one of first priority.

## Introduction

Malaria, a potentially life-threatening infectious disease, can become even more catastrophic when complicated by sepsis and septic shock. When patients present in such a critical condition, the currently available literature allows a dilemma to develop as to which the correct treatment strategy is concerning fluid resuscitation. On one hand a volume-restricted strategy is favoured in order to prevent pulmonary edema whereas on the other hand, guidelines for treatment of septic patients favour the opposite approach of aggressive volume replacement. Therefore, there seems to be a therapeutic conflict about how patients should be treated primarily. The present case demonstrates a patient with severe malaria and concomitant septic shock. In the discussion the authors explain their decision to focus the treatment towards sepsis and describe the steps taken until the patient's full recovery.

## Case presentation

A previously healthy 55-year-old Caucasian man from Germany was admitted to the emergency room with high fever (39.5-40°C), shivering and confusion. As an embassy member in a sub-Saharan African country, he left Africa one month prior to admission to our hospital. Our physical examination revealed a pulse of 150/min and a systolic blood pressure of 90 mmHg. Auscultation of the chest revealed ubiquitous wheezing and normal heart sounds without presence of murmurs. The skin was dry, but with no erythema. The abdomen was soft with no rebound tenderness. The liver was not enlarged and the spleen was not palpable. Neurological examination showed no focal neurological signs and no signs indicative of meningitis. The Glasgow Coma Scale was 11. Laboratory evaluation revealed a free hemoglobin of 48.2 μmol/l, total bilirubin of 59 μmol/l and lactate dehydrogenase of 16.8 μmol/l*s, indicative of ongoing hemolysis. Procalcitonin and C-reactive protein (CRP) were markedly elevated, and the patient showed a severe thrombocytopenia. Lactate was 7.5 mmol/l, base excess-4.5 mmol/l, anion gap 16.6 mEq/l and pH 7.43, indicative of a compensated metabolic acidosis. The presence of malaria falciparum parasites could be detected by microscopical examination of a thick blood film. The chest X-ray was normal. The patient received 500 ml of cristalloids and 1 gm paracetamol intravenously and was admitted to the intensive care unit (ICU).

In the ICU, a central venous catheter was inserted into the right jugular vein and a catheter for pulse contour analysis and continuous hemodynamic measurement was placed into the left femoral artery. The patient received colloids, crystalloids and a continuous infusion of glucose 10% under concurrent laboratory control of glucose, arterial blood gases, lactate and electrolytes every 4 hours. Two units of platelets were transfused. Quinine (QuinimaxR) was administered intravenously, starting with a bolus injection followed by continuous infusion over 24 hours under regular control of quinine blood levels. Additionally doxycycline was given orally. The first hemodynamic measurement revealed a central venous pressure (CVP) of 11 mmHg, cardiac output of 6.0 l/min and a systemic vascular resistance (SVR) of 729 dynes*s/cm2. CVP was measured every 4 hours. During the first 14 hours in the ICU, the overall fluid administration was 4.7 l and diuresis was 3.6 l, resulting in positive fluid balance of 1.1 liter. The heart rate decreased to 120 beats/min and the systolic blood pressure (SBP) increased to 100 mmHg with a mean arterial blood pressure (MAP) of 70 mmHg. The next day, the parasite load of 25.5% on admission increased to 36.7%. A second venous catheter was inserted into the right femoral vein and a red cell exchange with a cell seperator (Cobe Spectra, Gambro BCT, Munich, Germany) was initiated. A 1.5 fold blood volume red cell exchange was performed with 20 units of packed red blood cells. The procedure was well tolerated and no bleeding occurred. The parasite load dropped to 16%. The following day the hemodynamic status of the patient destabilized with a drop of SBP to 80 mmHg and of the MAP to 45 mmHg, respectively. The heart rate increased to 180 beats/min and the CVP was 3 mmHg. The respiratory rate was 25/min under supplementation of 10 l O2 given via venturi face mask. Laboratory data showed a peak lactate level of 21.4 mmol/l, a base excess of -16.4 mmol/l and a pH of 7.24. The patient showed severe agitation. A mechanical ventilation was immediately initiated under analgesia and sedation with propofol and sufentanil. The patient was breathing spontaneously by means of pressure support of 15 to 20 mmHg and a positive endexspiratory pressure of 5 mmHg. The shock was treated with fluid resuscitation according to the concept of early goal directed therapy. During the next 6 hours, administration of 2 liters of cristalloids and 1 liter of colloids was required to increase the CVP. Increase of MAP over 65 mmHg and a sufficient central oxygen venous saturation could only be reached by means of dobutamine and norepinephrine. After hemodynamic stabilization, CVP was around 12 mmHg. Lactate levels fell gradually to 5.5 mmol/l and the pH normalized. The net fluid balance was 5.3 l. Because of the high parasite load of 16%, a second red blood cell exchange via cell separation was undertaken with 13 units of packed red blood cells. The parasite load decreased to 2% the next day. The patient remained hemodynamically stable during the procedure with no need for increased doses of catecholamines. A CT scan of the brain showed no signs of bleeding, ischemia or edema. Due to increased myoglobin levels and the subsequently developed renal failure, renal replacement therapy was initiated via continuous venovenous hemodiafiltration (CVVHDF). Although the thrombocyte count remained low, administration of heparin was initiated at 200 i.u. per hour in order to prevent clotting of the dialysis machine and provide prophylaxis against deep vein thrombosis. As a consequence of the shock, the patient developed a severe hepatic dysfunction with a massive increase of liver enzymes and bilirubin and a decrease of albumin to one third of normal levels. Furthermore, the patient developed rhabdomyolysis. With a positive fluid balance of almost 4 liters the fourth day, the patient remained hemodynamically stable. Thereafter dobutamine could be stopped and norepinephrine slightly reduced. With a CVP between 9 and 13 the patient developed no pulmonary edema and all respiratory parameters remained stable. On the fifth day C-reactive protein (CRP) and leucocyte count increased, but temperature remained stable between 38 and 39°C. Cultures from blood, respiratory secretions and urine were performed and the patient was given antimicrobial treatment with a fixed preparation of combined piperacillin and tazobactam. All cultures remained negative. During the following days norepinephrine was further decreased based under hemodynamic status and was finally discontinued on the seventh day. Based on CVP measurements, the net fluid balance was negative from day 5 on until discharge from the ICU. Mechanical ventilation was discontinued on day 7, and continuous dialysis was replaced by intermittent dialysis. The extremely high scores (Acute Physiology and Chronic Health Evaluation II, Simplified Acute Physiology Score II, Sequential Organ Failure Assessment) on the day of the shock improved gradually. Hepatic dysfunction and rhabdomyolysis dissolved, but the patient remained in renal failure. Thrombocytes and leucocytes returned to normal values. After extubation the patient's confusion improved slowly. At discharge from the ICU on day 12, there was sufficient orientation in time and person, but not in situation. The patient remained hospitalized for a further period of three weeks. During this time he regained full orientation. At the time of discharge creatine level was 286 μmol/l, renal function had been resumed and thus there was no need for further dialysis.

## Discussion

At the time of presentation in our emergency room, the patient suffered from severe malaria. He was severely confused, but not comatosed, and during the first two days his hemodynamic and respiratory status remained stable. Despite of his stable status, the patient showed biochemical features indicating a poor prognosis [1], amongst others, hyperlactatemia, hyperbilirubinemia, elevated liver enzymes, and a very high parasite load. Procalcitonin, which was found to be pathological during or even prior to sepsis, was extremely high on admission in the ICU. After two days the patient developed a shock. The frequency of shock on admission to a hospital in patients with severe malaria is 7.7% [2], and is up to 21.5% in hospitals with ICUs, that are specialized in the treatment of infectious diseases [3]. In a study of 560 patients the overall mortalitiy of those patients with severe malaria was 14.8%. Fifty-six patients (10%) in that study died from an intractable shock [2]. In another study undertaken by Bruneel et al., 14 of 50 patients with severe falciparum malaria developed shock (28%). Seven of these patients died (14%). Once patients with severe falciparum malaria develop shock, the mortality rate ranges from 50 to 67.5% [4]. The cause of death in severe falciparum malaria is often multi-factorial, but as mentioned above shock is one of the leading causes, a fact that forces us to adapt a quick and sophisticated treatment approach towards the critically ill patient. But the question remains of how should a patient with severe falciparum malaria and shock be treated?

Discrepancies exist among the treatment recommendations by experts specialized in the treatment of tropical diseases and authors specialized in intensive care medicine. This might lead to a conflict about which treatment regimen would result in the best chances for patient survival from malaria complicated by shock, and what pitfalls in treatment should be considered.

Following the recommendations focused on the treatment of severe malaria [5,1], the patient should receive fluid replacement, which however that should be given with great caution, as the "dividing line" between overhydration and underhydration seems to be thin. The authors emphasize that circulatory overload caused by intravenous fluid administration, might precipitate acute lung failure as a consequence of pulmonary edema. They recommend that the central venous pressure should not be allowed to exceed 5 cm of water. In persisting hypotension dopamine was recommended as the catecholamine of choice.

Following the recommendations focused on the treatment of sepsis [6,7], fluid resuscitation should be started as soon as the sepsis syndrome has been recognized to reverse tissue hypoperfusion. Therapy goals include a CVP between 8 and 12 mmHg, MAP above 65 mmHg, a central venous oxygen saturation above 70%. CVP should be adjusted to a target between 12 and 15 mmHg in mechanically ventilated patients. If the goals cannot be achieved, transfusion of packed red blood cells and administration of dobutamine as the primary catecholamine to increase cardiac output are recommended.

We were aware, that the definition of septic shock refers to patients with bacterial infections, but nevertheless, the clinical, laboratory and hemodynamic signs in this case were the same as in patients with a shock as a result of a bacterial infection. We decided to treat our patient according to the early-goal directed strategy and the surving sepsis campaign guidelines. Our decision was based on several points: First, septic shock leads to circulatory abnormalities that result in tissue hypoxia, multiorgan failure and subsequent death [8]. Global tissue hypoxia in itself independently contributes to endothelial activation and disruption of the homeostatic balance, vascular permeability, and vascular tone [9]. These processes take place in all organs including the lungs. This may lead to acute lunge injury and to acute respiratory distress syndrome. The early goal-directed strategy, which includes rapid fluid resuscitation proved to have shown both short-term and long-term benefits. The benefits arose from restoration of sufficient oxygen delivery that reversed the above mentioned processes. Therefore restrain from aggressive fluid resuscitation to prevent a putative pulmonary edema, as suggested by "malaria recommendations", seems to be of no choice for our patient's treatment. Our strategy was supported by a study of Gachot et al., who showed, that acute lung injury is most often part of the multiple organ dysfunction syndrom in severe falciparum malaria [10]. Secondly, technical facilities of the ICU included mechanical ventilation performed with a lung-protected strategy [11], when acute lung injury occurred as a consequence of fluid overload. We weighed the risk of lung injury as a result of sepsis against respiratory failure as a result of fluid overload. The respiratory failure may result in the need for mechanical ventilation and bears the risk of acquiring ventilator-associated pneumonia. The risk of death in patients who require mechanical ventilation for more than 48 hours is between 15 to 50 % depending on the patientsś population studied [12,13]. The risk of acquiring ventilator-associated pneumonia seemed acceptable to us, since the risk of death as a consequence of insufficiently treated shock in patients with malaria outweighs the risk of death as a consequence of ventilator-associated pneumonia.

Thirdly, the fear of pulmonary edema from overhydration may be exaggerated in general, since Maitland et al. could demonstrate in children with severe malaria with or without anaemia, that volume resuscitation with albumin or saline does not lead to an increased risk of cardio-respiratory deterioration or pulmonary edema compared to the control group [14,15].

Lastly, more recent guidelines should give us recommendations based on a level of evidence. Surviving sepsis campaign guidelines [6] graded the early goal-directed therapy as level B, reflecting a data basis of one randomised, controlled and single centre study [7]. The data basis of recommendations for treatment of severe malaria and shock [5] is sparse. Although fluid restriction to a maximum CVP of 5 cm of water is strongly recommended, this is not supported by appropriate studies and thus it is not clear, where this recommendation comes from other than observational studies and case reports.

Taking all the above in consideration, we decided to treat our patient according to the sepsis guidelines. The patient survived, and despite having shown features indicative of a bad prognosis, such as the development of septic shock with multiorgan failure, he responded favourably to the undertaken treatment and was in a state of full recovery at the time of discharge. However, the patient presented is an isolated case that was used in order to discuss the currently available literature and the suggested recommendations. Furthermore, we discussed the case on the background of treatment options available in high-end ICU facilities. Treatment options and equipment availability is often entirely different in third world countries. Nevertheless, every treatment strategy should be based both on the respective clinical evidence and a pathophysiological concept and therefore, we believe that in patients with severe malaria complicated by septic shock, the treatment of sepsis and septic shock should be the one of first priority.

**Figure 1:**
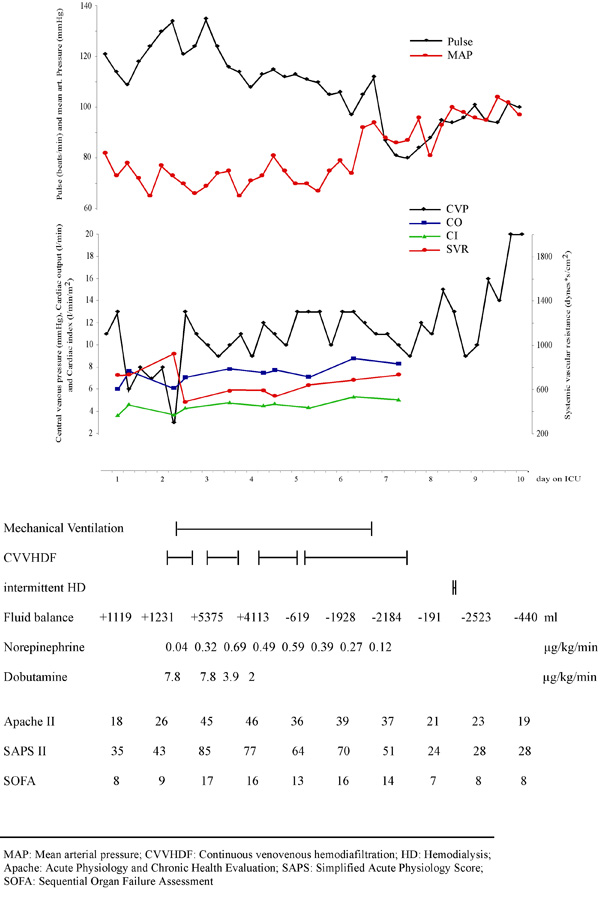
**The graphic is showing hemodynamic and circulatory data during the stay on ICU**. Below time course of mechanical ventilation, dialysis, fluid balance, treatment dose of catecholamines, and the sepsis scores are given.

**Table 1:** Laboratory data during stay on the intensive care unit

Day	1	2	3	4	5	6	7	8	9	10	11	12
Creatinine (72-127 μmol/l)	110	126	153	182	257	230	200	162	206	264	416	555
Urea nitrogen (3.0-9.2 mmol/l)	13.8	17.1	16.3	15	16.5	13.3	10.7	9.8	12.4	12.6	20.8	27.8
INR	1.1	1.2	1.4	1.4	1.3	1.1	1.1	1	1	1	1	1
Erythrocytes (4.5-5.9 Tpt/l)	4.3	3.3	4.3	3.6	3.5	3.5	3.3	3.3	3.6	3.6	3.6	3.5
Leucocytes (4.4-11.3 Gpt/l)	9.3	6.4	13.8	10.2	20.9	23.5	24.2	14.8	9.5	8.5	8.4	8.2
Thrombocytes (150-360 Gpt/l)	13	34	26	18	29	32	31	20	26	47	103	193
Hematocrit	0.39	0.3	0.37	0.31	0.31	0.31	0.31	0.29	0.32	0.32	0.32	0.31
Hemoglobin (8.7-10.9 mmol/l)	8.4	6.6	8	6.6	6.5	6.4	6.2	6.2	6.8	6.7	6.6	6.6
Free Hemoglobin (<3.1 μmol/l)	48.2	20.7	17.4	22.1	15.5	10.3	7.5	6.6	7.8	5.4		
CRP (<7.5 mg/l)	198	249	130	102	200	177	98.7	75	55	63	99	98
Procalcitonin (<0.5 ng/ml)	60	83		43	40	26	19				4	
Creatine kinase (μmol/l*s)	7.1	17.8	10.4	11.6	21.7	21.8	38.9	23.1	10.4	3.5	1.9	1.2
Myoglobin (17-106 μg/l)	1230	646	1822	1985	7774	11026	18883	6479	2362	753	574	393
LDH (2-4 μ mol/l*s)	16.8	11.1	16.3	39.6	25.3	14.4	11.4	9.2	7.8		7.2	7.1
ASAT (0.08-0.3 μmol/l*s)	1.37	1.55	6.28	36.6	16.8	8.2	4.63	3.39	2.57	1.58	0.99	0.77
ALAT (0.08-0.37 μmol/l*s)	0.4	0.41	2.53	13.2	12.9	10.5	6.86	4.87	3.95	3.08	2.38	1.96
GLDH (<67 nmol/s*l)			66	11515	3084	751	253	192		240	208	181
Total Bilirubin (<21 μmol/l)	59	101	60	29	24	25	22	21	36	35	27	20
Conjugated Bilirubin (<3.4 μmol/l)		57.6	30.9	18	15.4		16.3	13.9			21	15.1
Lactate (0.5-2.2 mmol/l)	7.5	3.8	18.2	5.6	2.9	1.6	1.2	1.0	2.1	1.2	0.6	0.6
Bicarbonate (16-22 mmol/l)	19.1	24.2	17.8	34	32	29.4	29	29.6	25.6	26.4	25.1	22.3
Base excess (−2.3-2.3 mmol/l)	−4.5	0.4	−5.7	10.1	7.1	4.2	4.7	6	2.4	3.2	0.8	−1.2

INR: normalized international ratio; CRP: C-reactive protein; LDH: Lactate dehydrogenase; ASAT: Aspartate aminotransferase; ALAT: Alanine aminotransferase; GLDH: Glutamate dehydrogenase

## Conclusion

On the basis of this original case of a patient with severe malaria complicated by a septic shock, we discussed two strategies of fluid resuscitation based on the currently available literature. We believe that the treatment of sepsis according to the sepsis campaign guidelines in these patients should obtain priority, since pathophysiological considerations as well as the evidence of available recommendations favouring this approach.

## List of abbreviations

ICU: Intensive Care Unit; CRP: C-Reactive Protein; CVP: Central venous pressure; SVR: Systemic vascular resistance; MAP: Mean arterial blood pressure; SBP: Systolic blood pressure; CT: Computer tomography; CVVHDF: Continuous venovenous hemodiafiltration.

## Consent

Written informed consent was obtained from the patient for publication of this case report. A copy of the written consent is available for review by the Editor-in-Chief of this journal.

## Competing interests

The authors declare that they have no competing interests.

## Authors' contributions

FK and RP performed the literature review. FK wrote the manuscript. RP, SR, KB and WP contributed to writing the manuscript. VK was a major contributor in writing the manuscript.
